# Preoperative anastomotic evaluation prior to ileostomy closure: A 5‐year UK survey, systematic review, and meta‐analysis

**DOI:** 10.1111/codi.70137

**Published:** 2025-06-12

**Authors:** D. Atraszkiewicz, T. Shakir, C. Harrington, P. Bassett, B. Soile, H. Mukhtar

**Affiliations:** ^1^ Broomfield Hospital Mid and South Essex NHS Foundation Trust Chelmsford UK; ^2^ University College London London UK; ^3^ Whittington Hospital Whittington Health NHS Trust London UK; ^4^ Statsconsultancy Ltd. Amersham Buckinghamshire UK

**Keywords:** anastomosis, assessment, closure, ileostomy, meta‐analysis, preoperative

## Abstract

**Aim:**

To compare current UK surgical practice against evidence‐based anastomotic evaluation techniques prior to ileostomy closure.

**Methods:**

An online survey was distributed to UK consultant colorectal surgeons with Association of Coloproctology of Great Britain and Ireland affiliation to assess preoperative investigations. Data were collected at two timepoints: 2019 and 2024. A systematic review and meta‐analysis were performed utilising PRISMA guidelines. MEDLINE (PubMed), Embase and Education Resources Information Center databases were evaluated from inception to 27 March 2024. Inclusion criteria were adult patients (≥18 years), distal colonic/pelvic anastomosis and defunctioning ileostomy reversal. ROBINS‐I bias assessments were conducted. DerSimonian and Laird random‐effects analyses were performed on eligible sensitivity and specificity data with forest plots generated. PROSPERO ID: CRD42024520236.

**Results:**

The survey received 221 (41.0%) and 212 (40.7%) responses in 2019 and 2024 respectively. Pre‐ and post‐pandemic practice was consistent. Water‐soluble contrast enema (WCE) and digital rectal examination (DRE) were the most utilised, performed ‘always’ by 83.2% and 78.7% respectively. Thirty‐seven studies (5061 patients) were included for systematic review; 12 studies (1385 patients) for meta‐analysis. Studies were heterogeneous in methodology; no randomised controlled trials were identified. Endoscopy showed higher sensitivity (73.1%) compared to retrograde contrast studies (WCE and pouchography; 53.1%) in identifying anastomotic leaks. Specificity was similar: 100% and 98.0% respectively. Significant heterogeneity and a lack of eligible studies limited further interpretation. CT has a limited evidence base for anastomotic evaluation.

**Conclusions:**

The most commonly performed anastomotic evaluation methods in the UK are WCE and DRE. Endoscopy, however, has a greater sensitivity and specificity for identifying anastomotic complications. WCE is an effective option to confirm suspected leaks. Endoscopy should be considered to evaluate anastomotic integrity prior to ileostomy closure.

**What does this paper add to the literature?**

There are currently no guidelines for optimal preoperative anastomotic assessment. To aid surgical decision making, this paper: (i) identifies current surgical anastomotic evaluation practice nationally; and (ii) reviews and analyses current evidence for these techniques.

## BACKGROUND

Complications from low rectal anastomoses are among the most daunting challenges colorectal surgeons face, often mitigated by a temporary ileostomy. Benign conditions, namely inflammatory bowel disease involving an ileal pouch–anal anastomosis (IPAA), present unique complexities. This is emphasised through the Ileoanal Pouch Report (2017), highlighting that 81.4% of IPAAs had diverting ileostomies [1].

However, within the UK, most ileostomies are performed concurrently with low anterior resections. In the National Bowel Cancer Audit Annual Report (2022), 7486 patients were diagnosed with rectal cancer between 1 April 2020 and 31 March 2021; 60% of low anterior resections received an ileostomy at the time of the index operation [2]. This is particularly pertinent as colorectal cancer is now the third most prevalent cancer and second highest cause of cancer‐related mortality in the UK [3, 4]. A year‐on‐year high, 47.4% of patients had unclosed ileostomies at 18 months post‐index surgery [2]. With data indicating a negative quality of life impact and even a risk factor for long‐term survival [5], local quality improvement targets aim to reduce the time interval to ileostomy reversal.

Current anastomotic evaluation methods include digital rectal examination (DRE); fluoroscopy with anterograde or retrograde water‐soluble contrast (WCE); CT with or without contrast enema; and direct visualisation with endoscopic evaluation. Often one, or a combination, of these investigations are performed. Additionally, after diagnosis of anastomotic leak (AL), follow‐up assessment is important.

However, there are currently no robust recommendations nor any existing meta‐analyses regarding which evaluative modality to perform. With 3810 ileostomy closures and 2178 colostomy closures performed in England in 2022–2023 [6], guidance for preoperative anastomotic evaluation is needed. This review therefore aims (i) to survey practice of UK consultant colorectal surgeons and (ii) to analyse current literature investigating anastomotic evaluation techniques prior to ileostomy closure.

## METHODS

### National survey

A six‐question electronic survey with 5‐point Likert scales was designed by the research group and supervising author using the web‐based hosting site Survey Monkey®. Survey recipients were asked to self‐report their preferences in performing (a) fluoroscopic contrast enema; (b) CT with contrast enema; (c) CT without contrast enema; (d) preoperative endoscopic evaluation; (e) DRE; and (f) concurrent on‐table endoscopic evaluation at the time of reversal (Appendix S1).

The survey was distributed in two phases, 5 years apart, to consultant colorectal surgeons with active Association of Coloproctology of Great Britain and Ireland (ACPGBI) affiliation. To prevent duplicated reporting of practice within surgical teams, only consultant surgeons were invited to the survey. Recipients were cross‐verified for having a searchable active National Health Service (NHS) email account. Invitation and reminder emails were sent. Phase one data were collected between 28 November 2019 and 3 January 2020 and phase two data were collected between 23 July 2024 and 1 September 2024. The phase two survey was created using Microsoft® Forms and included a seventh question that quantified the number of procedures performed per year (Appendix S2). The two surveys' methodology was otherwise identical. Distributing the survey at these two timepoints improved reliability of data collected and helped establish potential trends over the pre‐ and post‐pandemic periods. An ‘opt‐out’ option was also included to ensure voluntary participation. Those who opted out, had returnable emails, or had inactive NHS accounts were excluded from analysis.

### Systematic review

Three electronic databases were systematically evaluated in accordance with PRISMA guidelines [7] from their inception to 27 March 2024: MEDLINE (‘PubMed’), Embase and Education Resources Information Center (ERIC). The search strategy comprised keywords and synonyms including ‘ileostomy’, ‘reversal’, ‘closure’, ‘endoscopy’, ‘colonoscopy’, ‘sigmoidoscopy’, ‘computed tomography’, ‘digital rectal examination’, ‘contrast enema’ and ‘Gastrografin enema’. Additionally, a reverse‐citation search was performed on any included studies to review pertinent research not already included. This review was registered with PROSPERO; registration ID CRD42024520236.

Screening was independently performed by two reviewers in parallel. Conflicts were resolved during a consensus meeting with the whole research team. Duplicate records were removed and abstracts screened to exclude conference abstracts, review articles, case reports and correspondence articles. Full‐text review was performed to identify studies investigating evaluation techniques prior to ileostomy closure. Inclusion criteria were adult patients (≥18 years), distal colonic/pelvic anastomosis and defunctioning ileostomy reversal. No restriction to anastomotic type was made and no limitation was made to publication status.

Defined interventions included CT imaging with or without contrast enema, fluoroscopy with contrast (anterograde/enema), endoscopic evaluation and DRE. The comparator group (where applicable) was defined as having received no, or an alternative, preoperative investigation. Outcome measures were defined as anastomotic integrity assessed on intervention (leak/stricture/fistula), performance characteristics of the intervention if calculated (sensitivity/specificity/positive predictive value/negative predictive value), distal AL post‐reversal (radiological/biochemical/clinical diagnosis) and stoma status/outcome. Risk of bias assessment was performed using the Cochrane's Risk of Bias in Non‐Randomised Studies – of Interventions (ROBINS‐I) tool by two reviewers. No institutional review board approval was required for this study.

### Meta‐analysis

Articles were then screened for sensitivity and specificity data. Papers were only included if they recorded the raw values of true positive, true negative, false positive and false negative data. A Freeman–Tukey double arcsine transformation stabilised the variances before DerSimonian and Laird random‐effects analyses were performed. Heterogeneity was assessed by *I* ^2^ values (positive if above 50%) and chi‐squared tests. Forest plots were generated with uncertainty in calculated values signified via confidence intervals. Studies were weighted inversely to effect estimate variance. Certainty of evidence was assessed using a GRADE approach [8].

## RESULTS

### National survey

In phase one, 806 UK consultant colorectal surgeons were identified with 546 having searchable NHS emails. Surveys were successfully delivered to 539 surgeons and responses collected from 221 (41.0%). In phase two, 879 UK consultant colorectal surgeons were identified with 652 having searchable NHS emails. Surveys were successfully delivered to 521 surgeons and responses collected from 212 (40.7%). A minority of responses across both phases had questions skipped.

**FIGURE 1:**
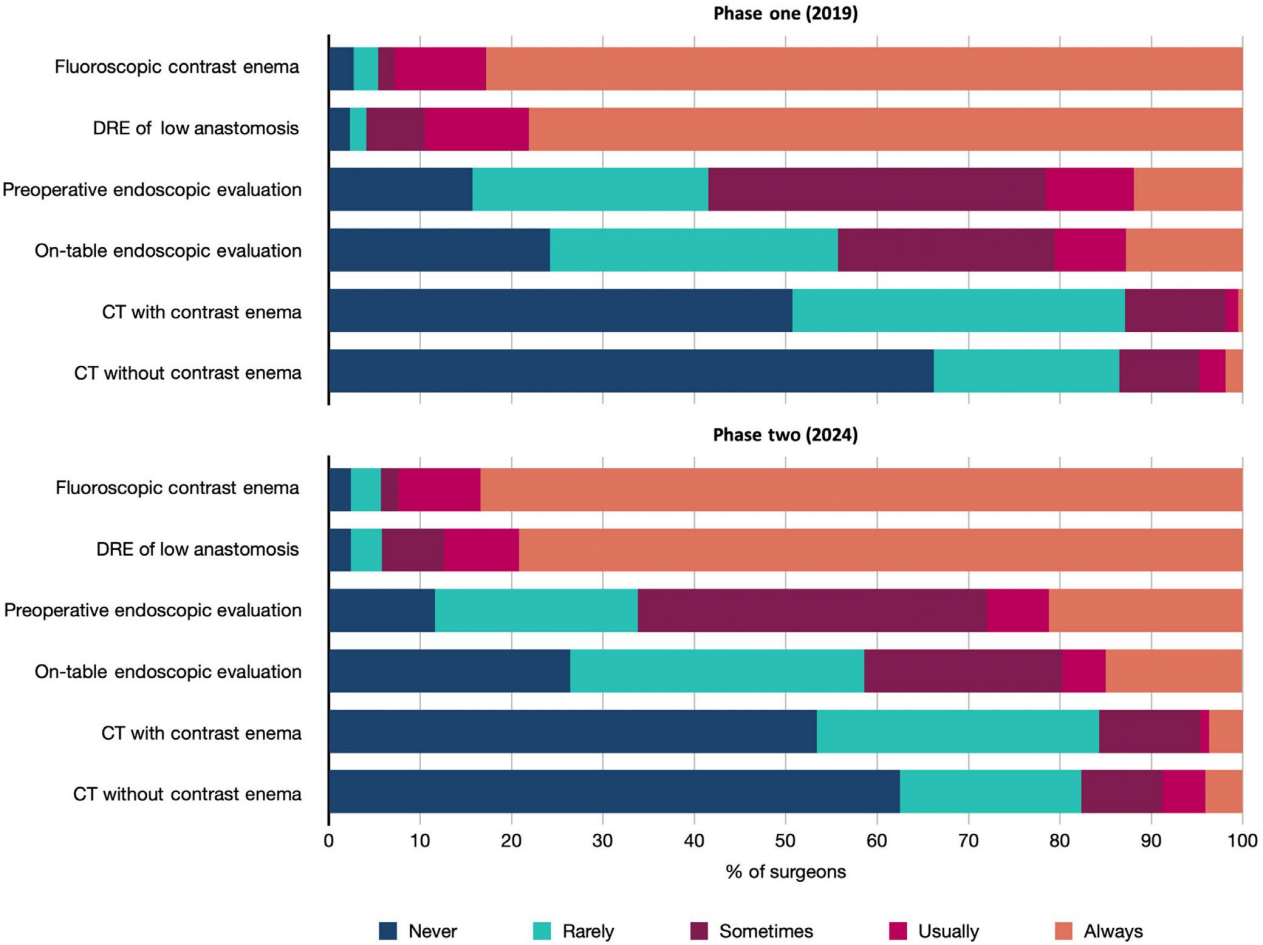
Bar charts showing the frequency of anastomotic evaluation techniques. Results are expressed as percentages and are ordered from most to least commonly performed.

Data collected across the two timepoints were grossly consistent; WCE and DRE were the most commonly reported methods of anastomosis evaluation prior to ileostomy closure (Table 1). WCE was either ‘always’ or ‘usually’ performed by 92.8% and 92.5% of consultant surgeons in 2019 and 2024 respectively. Similarly, DRE was either ‘always’ or ‘usually’ performed by 89.5% in 2019 and by 87.4% in 2024 (Figure 1). Endoscopic anastomotic evaluation was either ‘always’ or ‘usually’ performed preoperatively by 21.7% in 2019 and by 28.0% in 2024. On‐table use of the procedure was lower at 20.5% and 19.7% respectively. CT was consistently the least preferred modality. Most surgeons ‘never’ performed the procedure without contrast (2019 66.2%; 2024 62.5%) and with contrast (2019 50.7%; 2024 53.4%). Yearly rates of ileostomy reversals performed by phase two survey recipients were as follows: none (2; 0.9%), 1 to 10 (159; 75.0%), 11 to 20 (46; 21.7%), 21 to 50 (5; 2.4%) and >50 (0; 0%).

**TABLE 1:** Results from the national survey investigating anastomotic evaluation practices.

Investigation	Phase one (2019)					Phase two (2024)				
	Never	Rarely	Sometimes	Usually	Always	Never	Rarely	Sometimes	Usually	Always
Fluoroscopic contrast enema (*N*_2019_ = 221; *N*_2024_ = 212)	6 (2.7%)	6 (2.7%)	4 (1.8%)	22 (10.0%)	183 (82.8%)	5 (2.4%)	7 (3.3%)	4 (1.9%)	19 (9.0%)	177 (83.5%)
DRE of low anastomosis (*N*_2019_ = 220; *N*_2024_ = 207)	5 (2.3%)	4 (1.8%)	14 (6.4%)	25 (11.4%)	172 (78.1%)	5 (2.4%)	7 (3.4%)	14 (6.8%)	17 (8.2%)	164 (79.2%)
CT with contrast enema (*N*_2019_ = 209; *N*_2024_ = 191)	106 (50.7%)	76 (36.4%)	23 (11.0%)	3 (1.4%)	1 (0.5%)	102 (53.4%)	59 (30.9%)	21 (11.0%)	2 (1.0%)	7 (3.7%)
CT without contrast enema (*N*_2019_ = 207; *N*_2024_ = 192)	137 (66.2%)	42 (20.3%)	18 (8.7%)	6 (2.9%)	4 (1.9%)	120 (62.5%)	38 (19.8%)	17 (8.9%)	9 (4.7%)	8 (4.2%)
Preoperative endoscopic evaluation of anastomosis (*N*_2019_ = 217; *N*_2024_ = 207)	34 (15.7%)	56 (25.8%)	80 (36.9%)	21 (9.7%)	26 (12.0%)	24 (11.6%)	46 (22.2%)	79 (38.2%)	14 (6.8%)	44 (21.3%)
On‐table endoscopic evaluation of anastomosis (*N*_2019_ = 219; *N*_2024_ = 208)	53 (24.2%)	69 (31.5%)	52 (23.7%)	17 (7.8%)	28 (12.8%)	55 (26.4%)	67 (32.2%)	45 (21.6%)	10 (4.8%)	31 (14.9%)

*Note*: Phase one data collected between 28 November 2019 and 3 January 2020. Phase two data collected between 23 July 2024 and 1 September 2024. Total *N*_2019_ = 221. Total *N*_2024_ = 212.

Abbreviations: CT, computed tomography; DRE, digital rectal examination; *N*, number.

### Systematic review

**FIGURE 2:**
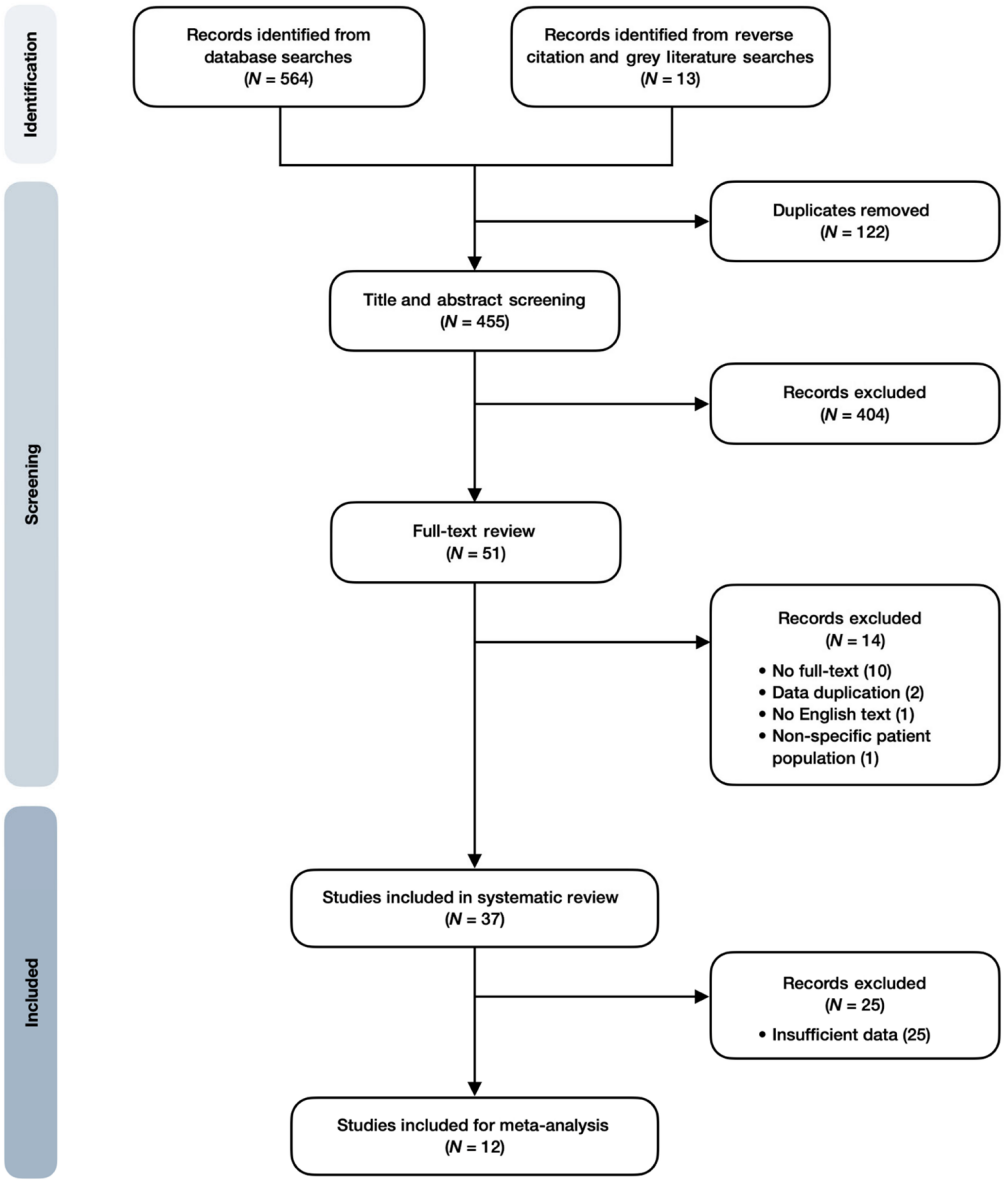
Flowchart of systematic search for studies investigating methods of evaluating anastomotic integrity prior to ileostomy closure.

Figure 2 shows results of the systematic search. Thirty‐seven studies—30 retrospective cohort (RC), four prospective cohort (PC), one case‐controlled (CC) and two non‐defined—were included (Table 2). These reported data for 5061 patients and spanned an evaluation period from 1981 to 2024. Three studies were multi‐centre. Fifty‐eight per cent (2944/5061) of patients underwent surgery for colorectal malignancy and 26% (1333/5061) for ulcerative colitis (UC); 47% (2394/5061) underwent an anterior resection; and 22% (1111/5061) underwent an IPAA.

**TABLE 2:** Characteristics of the 37 studies included within the systematic review.

Study	Design	Location	Multi‐centre	Disclosure	Evaluation timeframe	N patients	Patient population	Operation/anastomosis
Bakx 2003 [12]	PC	Amsterdam, ND	–	Grant × 1	–	27	Rectal cancer (22); trauma (1); other (4)	TME (22) [CAA]; rectosigmoid resection (4); primary (1)
Brown 1990 [9]	RC	Missouri, USA	N	–	–	18	UC (17); FAP (1)	IPAA (18)
Cai 2018 [13]	RC	Maryland, USA	N	Consult × 1	1994–2014	178	UC (170); IBD (8)	IPAA (178)
Cowan 2005 [14]	RC	Middlemore, NZ	N	–	1996–2003	59	Cancer (49); benign (10)	AR (59)
Da Silva 2004 [15]	RC	Florida, USA	–	Grant × 1	1990–2000	84	Rectal cancer (67); VA (11); stricture (4); RVF (2)	J pouch colorectal (84)
Dimitriou 2015 [16]	RC	Leicester, UK	N	N	2005–2010	339	Colorectal cancer (339)	Left colorectal resection (339)
Dolinsky 2007 [17]	RC	Philadelphia, USA	N	–	1998–2005	42	–	IPAA (42)
Exarchos 2019 [18]	RC	Athens, Greece	N	N	2010–2016	61	UC (61)	IPAA (61)
Farzaneh 2020 [19]	RC	California, USA	N	N	2014–2019	191	Rectal cancer (112); IBD (57); diverticular (7); other (15)	IPAA (59); J pouch ileorectal (1); J pouch colorectal (9); ileorectal anastomosis (2); CRA (120)
Goetz 2017 [44]	RC	Regensburg, Germany	N	N	2005–2011	252	Cancer (184); benign (71)	AR (15); TAR (94); TAR TME (16); TAR+ (7); intersphincteric res. rectum (8); rectum res. (18); colectomy (35); proctocolectomy (26); MVR (13); ileostomy (20)
Goh 2020 [20]	RC	Birmingham, UK	N	N	2009–2016	394	Cancer (260); other (133)	AR (394)
Gouya 2012 [45]	RC	Paris, France	–	–	2006–2010	195	Rectal endometriosis (195)	LAR (201); CAA (10)
Hong 2012 [21]	RC	Suwon, Korea	N	N	2005–2010	144	Rectal cancer (144)	LAR (144)
Horesh 2019 [22]	RC	Tel Aviv, Israel	N	–	2009–2018	116	Cancer (82); benign (34)	Low pelvic anastomosis (116)
Hrung 1998 [23]	RC	Philadelphia, USA	–	–	1988–1996	40	UC (37); FAP (3)	IPAA (40)
Jeyarajah 2008 [24]	RC	Leicester, UK	N	–	–	42	Rectal cancer (42)	AR (42)
Kalady 2008 [25]	RC	Wisconsin, USA	N	–	1999–2004	211	Rectal cancer (121); UC (56); other (34)	LAR [CRA/CAA] (121)
Karsten 2009 [26]	RC	California, USA	N	–	1998–2008	50	Rectal cancer (34); UC (4); FAP/HNPCC (3); other (9)	CRA (35); J pouch CAA (10); IPAA (5)
Katory 2017 [27]	RC	Gateshead, UK	N	N	2007–2013	45	Rectal cancer (45)	LAR (45)
Kelly 1994 [28]	–	London, UK	–	–	–	85	UC (75); FAP (7); CR cancer (2); constip (1)	IPAA (85)
Khair 2007 [29]	RC	Hull, UK	–	–	–	81	Rectal cancer (81)	LAR (81)
Killeen 2013 [30]	CC	Hull, UK	–	–	2005–2010	26	Cancer (26)	LAR (26)
Larsson 2015 [31]	RC	Helsingborg/Skane, Sweden	Y	–	2007–2009	95	Rectal cancer (91); benign (4)	LAR (95)
Lim 2006 [32]	PC	Leeds, UK	–	–	2000–2003	121	Rectal cancer/benign (121)	Low rectal anastomosis (121)
Lindner 2021 [33]	RC	Mannheim, Germany	Y	N	2005–2017	293	Rectal cancer (293)	LAR (293)
MacLeod 2004 [43]	PC	Aberdeen, UK	N	–	1999–2002	52	Rectal cancer (49); TVA (3)	LAR J pouch (52)
Malcolm 1995 [34]	RC	London, UK	–	–	1987–1992	25	–	IPAA (25)
Nabi 2013 [35]	RC	Bankstown‐Lidcombe, Australia	N	–	2008–2012	122	Colorectal cancer (90); UC (14); diverticular (12); other (6)	LAR (95); HAR (11); IPAA (14); subtotal (2)
Palmisano 2011 [36]	RC	Trieste, Italy	–	–	2003–2010	70	Rectal cancer (70)	CRA/CAA (70)
Santorelli 2018 [37]	RC	London, UK	N	N	2013–2015	61	UC (48); FAP (13)	IPAA [2‐stage] (20); IPAA [3‐stage] (40); redo‐IPAA (1)
Selvaggi 2012 [38]	RC	Naples, Italy	Y	N	1987–2010	186	UC (154); FAP (32)	IPAA (186)
Seo 2015 [10]	RC	Seoul, S. Korea	N	Grant × 6	2000–2012	163	Low rectal cancer (163)	LAR/uLAR (163)
Shalabi 2016 [39]	RC	Haifa, Israel	N	N	2006–2013	298	Rectal cancer (298)	LAR (298)
Sossenheimer 2019 [40]	RC	Chicago, USA	N	Grant × 1	2000–2010	262	UC (262)	IPAA (262)
Taylor 2021 [41]	RC	Melbourne, Australia	N	N	2012–2019	154	Colorectal cancer (154)	HAR (4); LAR (33); uLAR (116); IPAA (1)
Tsao 1992 [42]	–	Minnesota, USA	N	–	1981–1989	463	UC (428); FAP (35)	IPAA (463)
Worley 2021 [11]	PC	London, UK	N	N	2017–2019	16	UC (7); FAP (4); rectal cancer (5)	IPAA (11); CRA/CAA (5)

*Note*: Number in parentheses.

Abbreviations: AR, anterior resection; CAA, coloanal anastomosis; CC, case control; CRA, colorectal anastomosis; FAP, familial adenomatous polyposis; HAR, high anterior resection; IBD, inflammatory bowel disease (indeterminate); IPAA, ileal pouch–anal anastomosis; LAR, low anterior resection; MVR, multivisceral resection; PC, prospective cohort; RC, retrospective cohort; RVF, rectovaginal fistula; TAR, transanal resection; TAR+, transanal resection + one additional resection; TME, total mesorectal excision; TVA, tubulovillous adenoma; UC, ulcerative colitis; uLAR, ultra‐low anterior resection; VA, villous adenoma.

Investigation methods for pelvic anastomotic integrity included WCE [9, 10, 11, 12, 13, 14, 15, 16, 17, 18, 19, 20, 21, 22, 23, 24, 25, 26, 27, 28, 29, 30, 31, 32, 33, 34, 35, 36, 37, 38, 39, 40, 41, 42]; endoscopy [13, 18, 19, 22, 26, 27, 31, 33, 41, 43]; small bowel follow‐through (SBFT) (fluoroscopy with anterograde contrast via efferent stoma limb) [9, 19, 44, 45]; CT (anterograde or enema) [9, 19, 22, 45], MRI enema [11]; DRE/examination under anaesthesia [26, 31, 37, 39]; proctoscopy [31, 39]; and X‐ray pelvis [34].

The ROBINS‐I assessment [46] highlighted no studies with critical levels of bias; however, eight studies had ‘serious’ levels of bias (Appendix S3). This was primarily due to low patient enrolment (below 30 participants) [9, 11, 12, 30, 34]; prolongation of recruitment intervals [33]; exclusion of patients with early postoperative complications [14]; and investigations not performed on all enrolled patients [32].

#### Water‐soluble contrast enema

In 35 (94.6%) studies, WCE was evaluated to some degree. The mean time between primary surgery and fluoroscopy ranged between 1 and 34 weeks (Table 3). Radiological leak rates with WCE differed significantly across non‐IPAA cases from 0% [43] to 40% [27].

**TABLE 3:** Results of the 37 studies included within the systematic review.

Study	*N* patients	Female (%)	Mean age (years)	Anastomotic evaluation technique	Interval delay to evaluation^a^	Clinical leaks^b^ (%)	Radiological leaks (%)	Diagnostic strictures (%)	Diagnostic fistulas (%)	Post‐reversal leaks (%)
Bakx 2003 [12]	27	29.6	60	WCE	1	7.4	4.8	–	–	0
Brown 1990 [9]	18	61.1	36.0–37.0	SBFT; WCE; CT	4	–	12.5	–	–	–
Cai 2018 [13]	178	49.4	43.9–47.1	WCE: EE	–	–	–	–	–	–
Cowan 2005 [14]	59	33.9	70.0–72.0^c^	WCE	20–21^c^	–	5.7	14.3	–	0
Da Silva 2004 [15]	84	35.7	66.8	WCE	6–12	3.6	1.2	1.2	1.2	3.6
Dimitriou 2015 [16]	339	–	–	WCE	–	–	7.1	–	–	–
Dolinsky 2007 [17]	42	52.0	44.0	WCE	–	–	–	14.3	–	–
Exarchos 2019 [18]	61	36.0	38.2	WCE; EE	–	3.3	0.0	–	–	1
Farzaneh 2020 [19]	191	42.9	48.9–54.1	WCE; CT; SBFT; EE	–	–	–	3.7	–	1.4
Goetz 2017 [44]	252	38.0	59.5^c^	SBFT	–	–	5.5	–	1.5	–
Goh 2020 [20]	394	39.0	60.0	WCE	–	–	7.4	2.5	0.8	–
Gouya 2012 [45]	195	100	32.7	CT; SBFT	1	–	5.1	–	–	0.6
Hong 2012 [21]	144	42.4	59.5	WCE	34	–	0.7	3.5	–	2.1
Horesh 2019 [22]	116	44.0	61.0	WCE; EE; CT	43.5	–	–	–	–	4.3
Hrung 1998 [23]	40	37.5	34.0	WCE	8–12	5.0	12.5	–	–	–
Jeyarajah 2008 [24]	42	31.0	68.0^c^	WCE	6–8	–	28.9	2.6	–	0
Kalady 2008 [25]	211	40.3	53.2	WCE	15.8	3.3	–	–	–	0.05
Karsten 2009 [26]	50	45.0	50.8–55.2	WCE; EE; DRE	–	6.0	–	5.3	–	0
Katory 2017 [27]	45	22.0	66.0^c^	WCE; EE	8–12	–	40.0	–	–	–
Kelly 1994 [28]	85	–	38.0	WCE	5–6	–	9.4	4.7	–	–
Khair 2007 [29]	81	35.8	70.0^c^	WCE	22	3	5.8	–	–	–
Killeen 2013 [30]	26	30.8	72.0^c^	WCE	–	–	50.0	–	–	–
Larsson 2015 [31]	95	40.0	65.2	WCE; DRE; EE	–	9.5	12.0	–	–	3.2
Lim 2006 [32]	121	–	–	WCE	12	8.3	8.3	7.2	–	–
Lindner 2021 [33]	293	34.0	62.0^c^	WCE; EE	–	29.4	5.1	–	0.3	2.0
MacLeod 2004 [43]	52	48.1	64.0^c^	WCE	16.9	0	0	0	0	0
Malcolm 1995 [34]	25	48.0	38.0	WCE; XRP	11	–	32.0	–	–	–
Nabi 2013 [35]	122	38.5	43.0	WCE	14.9	9.0	6.6	9.4	–	–
Palmisano 2011 [36]	70	–	–	WCE	2	17.1	24.3	–	–	–
Santorelli 2018 [37]	61	41.0	39.0	WCE; EUA	14	11.5	1.9	–	–	–
Selvaggi 2012 [38]	186	54.8	36.9–39.8	WCE	–	–	0	0	0	1.6
Seo 2015 [10]	163	–	–	WCE	21.4	1.6	6.3	–	–	0
Shalabi 2016 [39]	298	41.0	62.2	WCE; DRE; P	–	3.2	0.0	3.7	–	0
Sossenheimer 2019 [40]	262	40.0	36.4	WCE	–	7.6	10.3	15.3	–	–
Taylor 2021 [41]	154	26.0	65.0^c^	WCE; EE	–	3.9	3.8	7.6	–	–
Tsao 1992 [42]	463	47.7	32.0	WCE	–	–	7.1	9.3	–	–
Worley 2021 [11]	16	37.0	39.0^c^	WCE; MRE	20	43.8	31.3	–	6.3	0

Abbreviations: CT, computed tomography (anterograde or enema contrast); DRE, digital rectal examination; EE, endoscopic evaluation (including flexible sigmoidoscopy/pouchoscopy/rectoscopy); EUA, examination under anaesthesia; MRE, magnetic resonance enema; P, proctoscopy; SBFT, small bowel follow‐through; WCE, water‐soluble contrast enema (fluoroscopy); XRP, X‐ray pelvis.

^a^ Mean duration (weeks) from anastomosis formation to formal anastomosis evaluation pre‐reversal.

^b^ Anastomotic leaks post primary operation.

^c^ Median.

RC studies reported varying sensitivities for WCE in identifying AL, from 0% [15] to 100% [27]. A PC study, with a 4.8% radiological leak rate, described routine WCE use 1‐week post primary surgery to assist early ileostomy takedowns [12]. None of these 18 cases (median stoma takedown 11 days) suffered a distal AL postoperatively. An RC of 2111 patients concluded that WCE does not influence clinical management and suggested that occult leaks are principally inconsequential while significant leaks will become clinically apparent [25]. One RC study highlighted that WCE was not beneficial prior to ileostomy reversal [14], while another PC study described no benefit from routine WCE but recommended adoption in the early postoperative period to improve anastomotic salvage rates if suggestion of AL [43].

Among 191 pelvic anastomoses evaluated with either endoscopy or combined endoscopy with contrast studies (WCE/CT/SBFT), no significant differences in clinical outcomes were demonstrated post‐reversal, including anastomotic complications, pelvic abscesses and readmission rates [19]. Full concordance in identifying AL was also seen between WCE, DRE and endoscopy in a CC study [31]. WCE showed a false positive AL identification rate of between 21.2% [24] and 28.9% [27]. Inter‐observer concordance among radiologists of 83% was depicted, with false positives often due to ‘dog‐eared’ anastomotic appearances from multiple laparoscopic stapler firings intra‐corporeally [27].

Radiological AL rates varied between cancer and benign cases (10.4% and 1.5% respectively) [20]. Seventy per cent of radiological leaks in one study experienced immediate postoperative complications and longer hospital stays (mean difference 6.8 days) [16]. Significantly higher delayed WCE leak rates were seen in patients with previous clinical AL, 66.7% [36] and 32.4% [10] compared to 15.5% and 4.7% in patients without prior clinical leaks respectively.

Performing serial WCEs over a median of 17 months demonstrated a 100% healing rate (10/10) of occult radiological AL [32]. Only 30.8% (4/13) of previous clinical leaks healed. An RC study of 163 patients with low rectal cancer demonstrated a 20% spontaneous AL healing rate on serial WCE [10]. However, 81.8% of those reversed despite persistent radiological AL had no future anastomotic‐related issues. Additionally, 42.9% of those with a delayed radiological AL on WCE after postoperative clinical AL could not have their stoma reversed, demonstrating the importance of interval WCE pre‐stoma reversal in this population.

#### Small bowel follow‐through (fluoroscopy and anterograde contrast)

Very few included studies evaluated the use of fluoroscopy with anterograde contrast via the efferent stoma limb. Using this method, local anastomotic complications can be evaluated [44]. The approach identified stenosis in the aboral stoma limb in 12% of examinations. Postoperative day eight SBFT had a sensitivity and specificity of 86% and 100% respectively for AL, but a poor sensitivity of 33% for pelvic abscesses [45].

#### CT colonography

A study assessed the use of CT colonography in early stoma reversal (8 days postoperatively) after complex endometriosis surgery, reporting a sensitivity and specificity of 100% for both AL and pelvic abscesses [45]. CT colonography also identified three (2.5%) patients with asymptomatic small bowel entrapments behind the descending mesocolon. These were corrected laparoscopically at the time of stoma closure. The corresponding SBFT in this group suffered significantly greater small bowel obstruction which may be related to the CT sensitivity for this pathology.

#### MRI enema

MRI enema demonstrated comparable, if not favourable, outcomes in diagnosing pelvic anastomotic defects compared to WCE [11]. MRI assisted in defining the radial location of the leak while furthering the anatomical detail compared to fluoroscopy. Performance characteristics of the investigation were not definable; however, MRI identified an additional AL (one of five) missed on WCE.

#### Endoscopic evaluation

Concordance between endoscopic and radiological contrast evaluations was demonstrated in 82 of 100 anastomoses [19]. Contrast studies failed to identify 11 anastomotic strictures and one sinus tract. Correspondingly, endoscopy failed to identify five strictures and one sinus tract highlighted radiologically. One RC study determined higher sensitivity of endoscopic evaluation for AL in previously symptomatic and asymptomatic patients (77% and 67% respectively) compared to WCE [37]. The study additionally determined that WCE provided no additional information.

#### IPAA subgroup

WCE was the principal anastomotic evaluation technique among patients post‐IPAA. The largest series of pouchograms post‐IPAA advocates for routine use [42]. Abnormal studies were associated with future pouch failure (23% vs. 6%), required stricture dilation under anaesthesia (33% vs. 4%) and had lower ileostomy closure rates (17% vs. 90%).

An RC study of 262 patients with UC demonstrated the predictive value of routine WCE; contrast extravasation was associated with a hazard ratio of 5.88 for future pouch failure [40]. Higher AL rates were revealed with fluoroscopy (anterograde/enema) in the symptomatic (2/9) versus asymptomatic (0/7) postoperative groups [9]. A silent leak rate of 8.1% (3/37) was also reported with IPAA [23]. However, two patients underwent reversal despite persistent radiological AL, suggesting false positives. The sensitivity of WCE for AL has also been described as low as 33.3% in a small population of 51 patients with three (6%) true leaks [37].

Another RC study evaluated the use of WCE for anastomotic strictures post‐IPAA, demonstrating sensitivity, specificity and false positive rates of 100%, 92% and 8.3% respectively [17]. In their stapled IPAA series, on WCE, a disrupted ileoanal staple ring had a 53.8% rate of AL and pelvic collection. In comparison, an intact ring had an 11.1% rate of AL and pelvic collection. Of those with WCE positive for AL, 62.5% developed a pelvic collection. In the negative WCE group, this was 23.5%.

An RC study evaluating pouchoscopy in 178 patients demonstrated no significant difference in early or late complications compared with WCE [13]. Among 84 patients post‐IPAA, pouchoscopy demonstrated a low diagnostic sensitivity for AL and stricture of 0% and 33.3% respectively [15]. Additionally, pouchoscopy and pouchograms in 61 UC patients demonstrated a specificity reaching 100% for WCE in evaluating AL [18].

### Meta‐analysis

**FIGURE 3:**
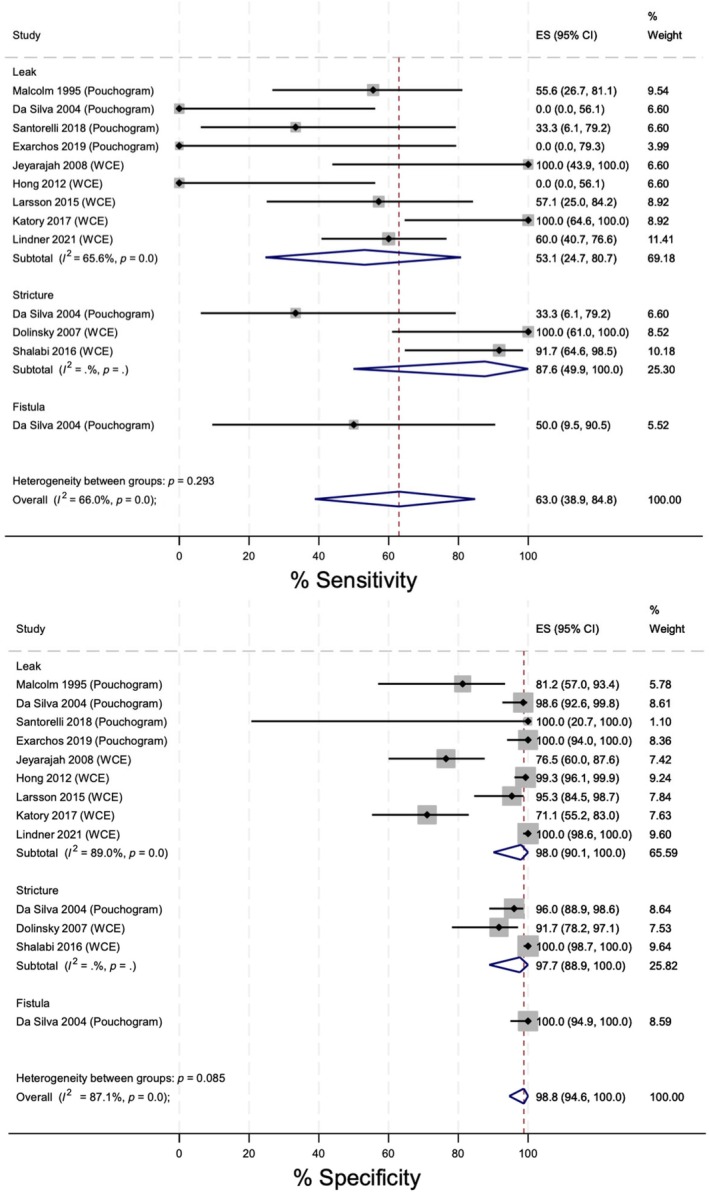
Forest plot showing the analysis of the sensitivity and specificity of retrograde contrast studies (WCE and pouchogram) in identifying anastomotic leaks, strictures and fistulas.

Twelve retrospective cohort studies (1385 patients) were included for analysis. Due to a paucity of data, a pooled analysis of retrograde contrast studies (WCE and pouchogram) was performed (Figure 3). Specificity (98.8%) was superior to sensitivity (63.0%) in identifying overall pathology (Table 4). WCE outperformed pouchography; however, there was insufficient power to perform separate subgroup analyses. Heterogeneity was significant for both leak rates and overall pathology rates, thereby limiting conclusions drawn.

**TABLE 4:** Summary of findings.

Investigation(s)	Pathology	*N* studies	*N* patients	Sensitivity (%)	Specificity (%)	Certainty (GRADE)^a^
Retrograde contrast studies (WCE and pouchography)	Anastomotic leak	9	735	53.1	98.0	Very low
	Stricture	3	432	87.6	97.7	Very low
	Fistula	1	74	50.0	100	Very low
	Overall	11	1089	63.0	98.8	Very low
Endoscopic procedures (rectoscopy, pouchoscopy and flexible endoscopy)	Anastomotic leak	3	420	73.1	100	Very low
Computed tomography (CT)	Anastomotic leak	1	118	100	100	Very low
	Abscess	1	118	100	100	Very low
	Overall	1	118	100	100	Very low
Digital rectal examination (DRE)	Anastomotic leak	1	93	57.1	100	Very low
	Stricture	1	312	100	100	Very low
	Overall	2	405	91.4	100	Very low
Small bowel follow‐through (SBFT)	Anastomotic leak	1	77	85.7	100	Very low
	Stricture	1	77	33.3	100	Very low
	Overall	1	77	72.7	100	Very low
Rigid proctoscopy	Stricture	1	312	100	100	Very low

Abbreviations: GRADE, Grading of Recommendations Assessment, Development and Evaluation; *N*, number; WCE, water‐soluble contrast enema.

^a^ As per reference 8. Interpretation: very low—we have very little confidence in the effect estimate; the true effect is likely to be substantially different from the estimate of effect.

**FIGURE 4:**
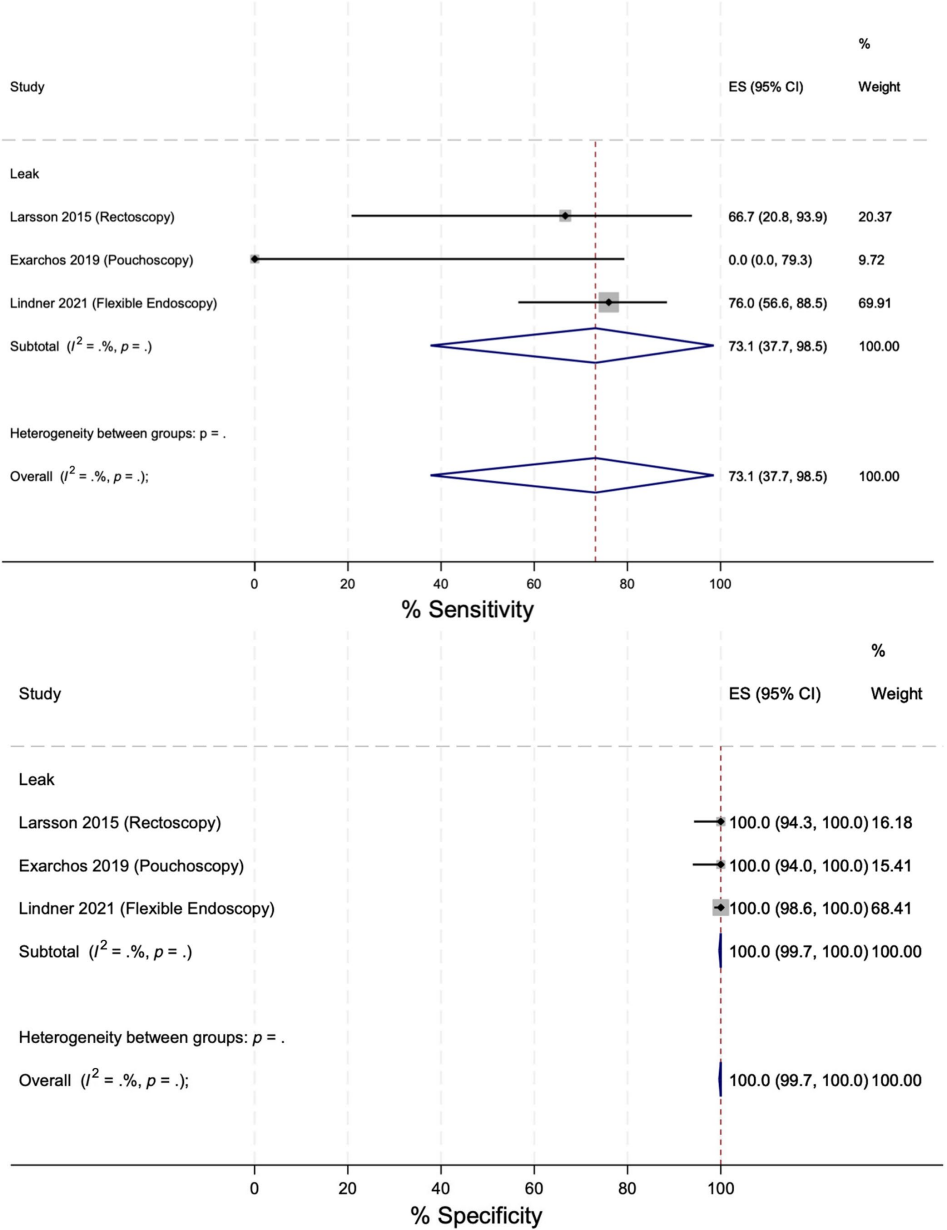
Forest plot showing the analysis of the sensitivity and specificity of endoscopic procedures (rectoscopy, pouchoscopy and flexible endoscopy) in identifying anastomotic leaks.

Due to a lack of studies, heterogeneity could not be assessed for the remaining analyses. Endoscopic procedures (i.e., rectoscopy, pouchoscopy and flexible endoscopy) showed higher overall sensitivity (73.1%) and specificity (100%) for identifying AL (Figure 4). Three studies were included in this analysis, limiting external validity.

Remaining analyses contained data for only one study per complication; therefore, limited conclusions could be drawn. Forest plots outlining sensitivity and specificity for each are outlined in Appendix S4. CT reported a higher sensitivity (100%) and specificity (100%) for identifying AL and abscesses compared to SBFT (sensitivity 72.7%; specificity 100%). The sensitivity and specificity of DRE in identifying AL and strictures were 91.4% and 100% respectively. Finally, rigid proctoscopy reported both a sensitivity and a specificity of 100% in identifying strictures.

There was an insufficient number of studies to create funnel plots; therefore, publication bias was not included in GRADE assessments. All findings were attributed ‘very low’ levels of certainty following downgrading for lack of randomised controlled trials, risk of bias and/or data inconsistency.

## DISCUSSION

The national survey demonstrated low variation in practice across both timepoints; however, there was significant variation in anastomotic evaluation between consultant surgeons. WCE and DRE were the most commonly reported preoperative methods of anastomotic evaluation, on average ‘always’ performed by 83.2% and 78.7% of surgeons respectively.

Survey results suggest endoscopy is performed six times fewer than WCE: on average, ‘always’ performed by 16.7% preoperatively and by 13.9% on‐table. This is despite recommendations favouring only endoscopic evaluation before reversal [23, 35]. Although our analyses showed superior sensitivity and specificity of endoscopic procedures in identifying AL compared to WCE and pouchography, all findings had ‘very low’ levels of certainty. This highlights the need for further research investigating the efficacy of these preoperative evaluation modalities. Endoscopy is time and resource intensive. Detailed cost–benefit analyses are required. Post‐pandemic pressures on healthcare systems are significant; COVID‐19 saw endoscopy activity reduce to 5% of pre‐pandemic levels [47]. Services continue to recover; as of November 2024, 42% of incomplete non‐urgent general surgery referrals have surpassed 18‐week targets [48]. It is important to emphasise that workup prior to ileostomy reversal remained grossly consistent pre‐ and post‐pandemic, despite associated clinical paradigm shifts.

No surgeons in either 2019 or 2024 reported ‘always’ performing all investigation modalities. One surgeon in 2024 (none in 2019) reported ‘never’ performing all anastomotic evaluation methods. Three respondents performed one investigation at least ‘rarely’ and ‘never’ performed all other investigations: one surgeon in 2019 who ‘always’ performed WCE, and two in 2024 who ‘always’ performed WCE and on‐table endoscopy respectively. In 2019 and 2024 respectively, 52 (23.5%) and 48 (22.6%) surgeons reported performing all six modalities at least ‘sometimes’.

The majority of studies included were small, retrospective, single‐centre and of heterogeneous methodology. Subsequently, meta‐analyses were limited due to heterogeneous and inconsistent data. Although a small number of studies advocated for routine WCE use [10, 30, 32, 35, 36, 41], most studies recommended selective use in high‐risk patients and/or to confirm and characterise leaks identified through other means [12, 14, 15, 16, 20, 22, 24, 25, 26, 29, 31, 39, 43]. Of persistent occult radiological leaks, 81.8% underwent uneventful ileostomy closure [10]. This highlights the likely high false positive rate with WCE and that delay from anastomotic evaluation to ileostomy closure could allow leaks to fully heal. Similarly conflicting reports for routine WCE in the IPAA population were described. Several studies support its routine use in this patient population [17, 23, 28, 40, 42] compared to advocates for its selective use [13, 18, 37, 38].

CT evaluation was scarcely reported to be performed; on average, ‘never’ performed by 64.4% without contrast and by 52.1% with contrast. CT colonography was recommended by one study as a screening tool for assessing low pelvic anastomoses [45]. Although not included in the national survey, MRI enema was concluded by one study to offer comparable outcomes in diagnosing pelvic anastomotic defects compared to WCE [11]. However, economic implications and performance characteristics compared to other cross‐sectional imaging modalities remain to be described.

Several patient care pathways have been suggested whilst awaiting stoma closure. One study recommended asymptomatic patients have evaluation with DRE and proctoscopy at 4–6 weeks and again at 12–14 weeks post primary procedure [25]. Following clinical concern, radiological examinations could be arranged. Overall, anastomotic evaluation closer to the reversal date was recommended. Routine WCE use was suggested on days 10–14 post primary surgery to identify leaks during a potential ‘therapeutic window of opportunity’ [30]. This highlights that patients with occult radiological AL had statistically significant higher pre‐closure investigations/procedures (mean difference 1), longer time to reversal (mean difference 6.2 weeks) and a mean extra cost of £1885/patient.

Practical considerations include that contrast enema may be more useful for patients with higher pelvic anastomoses, or a J pouch, whereas endoscopy and digital rectal examination may be more helpful in lower anastomoses. In addition, endoscopic evaluation may be more useful in identifying strictures and allow synchronous therapeutic interventions.

## LIMITATIONS

Surgeons registered with ACPGBI but working in the Republic of Ireland and those solely privately practising were excluded from the survey, possibly introducing selection bias. Additionally, the moderate response rates may have introduced response bias. Furthermore, Likert scale frequency outcomes lacked fixed time definitions allowing a degree of interpretation among participants.

No relevant randomised controlled trials were identified in this review, thereby limiting analyses performed. Comparing outcomes and performance characteristics of interventions remained challenging due to methodological heterogeneity. Further limitations of studies included small sample sizes; short follow‐up; heterogeneous definitions for AL and stricture; infrequent leak rates; and high levels of bias. Additionally, due to a shortage and heterogeneity of data, it was not possible to accurately interpret SBFT, rigid proctoscopy, CT, nor DRE analyses.

## CONCLUSION

The most commonly performed anastomotic evaluation techniques prior to ileostomy closure within the UK are WCE and DRE. Current literature suggests WCE has greatest benefit in high‐risk patients and to confirm leaks already identified clinically. Endoscopy was much less frequently performed; however, there may be greater sensitivity and specificity in identifying AL compared to retrograde contrast studies. CT (both with and without contrast enema) is the least used preoperative modality, with limited supportive evidence. Endoscopy and DRE should be considered preoperatively to evaluate anastomotic integrity prior to ileostomy closure. WCE may be performed to confirm identified leaks. Greater evidence, including cost–benefit analyses, is required to produce robust clinical guidance.

## AUTHOR CONTRIBUTIONS

**D. Atraszkiewicz:** Methodology; data curation; formal analysis; investigation; writing – original draft. **T. Shakir:** Writing – review and editing; methodology; formal analysis. **C. Harrington:** Data curation; writing – original draft. **P. Bassett:** Formal analysis. **B. Soile:** Methodology; data curation. **H. Mukhtar:** Conceptualization; writing – review and editing.

## FUNDING INFORMATION

No funding was received for this article.

## CONFLICT OF INTEREST STATEMENT

The authors report no conflicts of interest and that no financial support was received.

## ETHICS STATEMENT

Ethical approval was not required.

## Supporting information


Appendix S1.

Appendix S2.

Appendix S3.

Appendix S4.

## Data Availability

Data sharing is not applicable to this article as no new data were created or analyzed in this study.
