# Supplementary material for: Preoperative anastomotic evaluation prior to ileostomy closure: A 5‐year UK survey, systematic review, and meta‐analysis
Source: Colorectal Dis. 2025 Jun 12;27(6):e70137. doi: 10.1111/codi.70137 (PMC12159718; doi:10.1111/codi.70137)
Supplement: Supplementary file 1 — Appendix S1. [file CODI-27-0-s002.pdf]

Preoperative Anastomotic Evaluation Prior to Ileostomy Closure: A Five-Year UK Survey, Systematic Review, and Meta-Analysis

D. Atraszkiewicz [1], T Shakir [2,3], C. Harrington [3], P. Bassett [4], B. Soile [3], H. Mukhtar [2,3]

Supplement 1 — Phase One Survey

Figure 1: Screenshot of phase one (2019) national survey created and distributed via Survey Monkey®.

Investigations prior to reversal of temporary defunctioning stomas

Prior to reversal of a defunctioning stoma for a rectal anastomosis, do you perform:

1. Fluoroscopic contrast enema

- ○ Always
- ○ Usually
- ○ Sometimes
- ○ Rarely
- ○ Never

2. CT with a contrast enema

- ○ Always
- ○ Usually
- ○ Sometimes
- ○ Rarely
- ○ Never

3. CT without a contrast enema

- ○ Always
- ○ Usually
- ○ Sometimes
- ○ Rarely
- ○ Never

4. Endoscopic visualisation of the anastomosis preoperatively

- ○ Always
- ○ Usually
- ○ Sometimes
- ○ Rarely
- ○ Never

5. Digital rectal examination of low anastomosis

- ○ Always
- ○ Usually
- ○ Sometimes
- ○ Rarely
- ○ Never

6. On-table examination of the anastomosis with endoscopy

- ○ Always
- ○ Usually
- ○ Sometimes
- ○ Rarely
- ○ Never

Submit

Powered by
SurveyMonkey
See how easy it is to create a survey.
