# Supplementary material for: Preoperative anastomotic evaluation prior to ileostomy closure: A 5‐year UK survey, systematic review, and meta‐analysis
Source: Colorectal Dis. 2025 Jun 12;27(6):e70137. doi: 10.1111/codi.70137 (PMC12159718; doi:10.1111/codi.70137)
Supplement: Supplementary file 2 — Appendix S2. [file CODI-27-0-s004.pdf]

Preoperative Anastomotic Evaluation Prior to Ileostomy Closure: A Five-Year UK Survey, Systematic Review, and Meta-Analysis

D. Atraszkiewicz [1], T Shakir [2,3], C. Harrington [3], P. Bassett [4], B. Soile [3], H. Mukhtar [2,3]

Supplement 2 — Phase Two Survey

Figure 1: Screenshot of phase two (2024) national survey created and distributed via Microsoft® Forms. Of note, a seventh question was added which recorded the number of ileostomy reversals performed by recipients per year.

Preoperative anastomotic evaluation prior to ileostomy closure

Prior to the reversal of a temporary ileostomy for a distal anastomosis, do you perform:

1. CT *with* a contrast enema

- ○ Always
- ○ Usually
- ○ Sometimes
- ○ Rarely
- ○ Never

2. CT *without* a contrast enema

- ○ Always
- ○ Usually
- ○ Sometimes
- ○ Rarely
- ○ Never

3. Water-soluble contrast enema

- ○ Always
- ○ Usually
- ○ Sometimes
- ○ Rarely
- ○ Never

4. Digital rectal examination of low anastomosis

- ○ Always
- ○ Usually
- ○ Sometimes
- ○ Rarely
- ○ Never

5. Endoscopic evaluation of the anastomosis pre-operatively

- ○ Always
- ○ Usually
- ○ Sometimes
- ○ Rarely
- ○ Never

6. Endoscopic evaluation of the anastomosis on-table

- ○ Always
- ○ Usually
- ○ Sometimes
- ○ Rarely
- ○ Never

7. Approximately, how many ileostomy reversals do you perform every year?

- ○ None
- ○ 1 10
- ○ 11 20
- ○ 21 50
- ○ >50
