# Supplementary material for: Preoperative anastomotic evaluation prior to ileostomy closure: A 5‐year UK survey, systematic review, and meta‐analysis
Source: Colorectal Dis. 2025 Jun 12;27(6):e70137. doi: 10.1111/codi.70137 (PMC12159718; doi:10.1111/codi.70137)

Preoperative Anastomotic Evaluation Prior to Ileostomy Closure: A Five-Year UK Survey, Systematic Review, and Meta-Analysis

D. Atraszkiewicz [1], T Shakir [2,3], C. Harrington [3], P. Bassett [4], B. Soile [3], H. Mukhtar [2,3]

Supplement 3 — Risk of Bias Assessment

Figure 1: Traffic light plot summarising risk-of-bias (ROBINS-I) assessment for thirty seven studies included in systematic review.

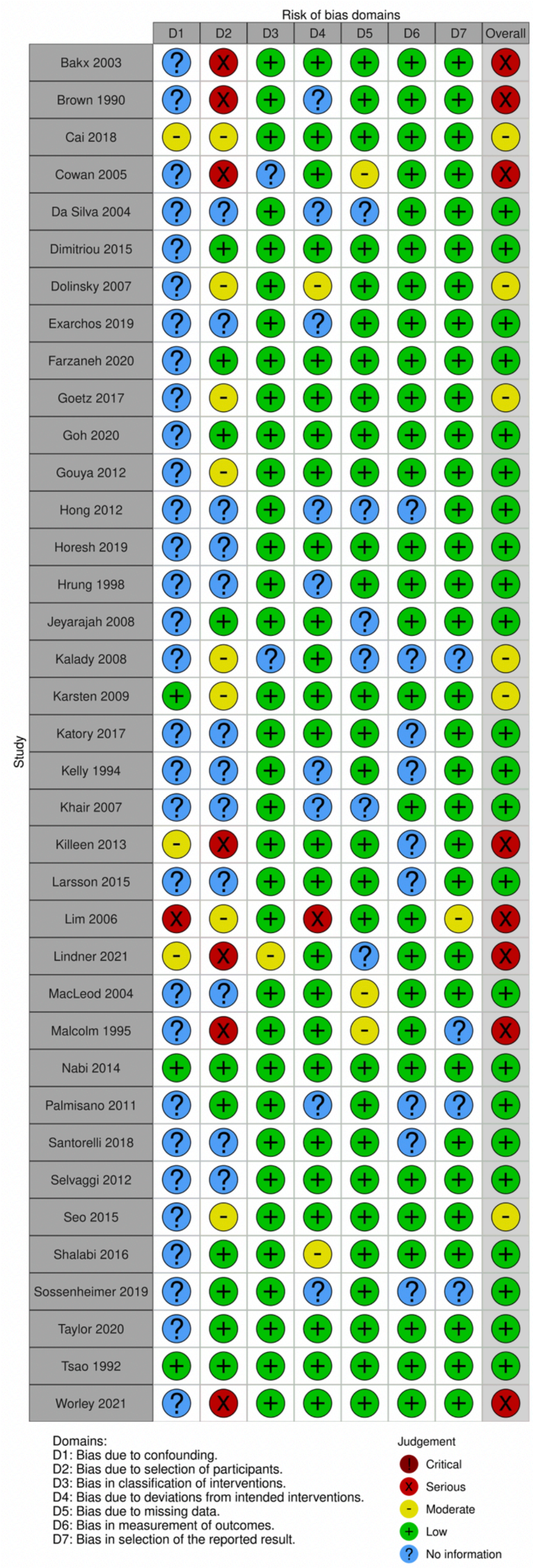

Supplement: Supplementary file 3 — Appendix S3. [file CODI-27-0-s003.pdf]
