# Supplementary material for: Preoperative anastomotic evaluation prior to ileostomy closure: A 5‐year UK survey, systematic review, and meta‐analysis
Source: Colorectal Dis. 2025 Jun 12;27(6):e70137. doi: 10.1111/codi.70137 (PMC12159718; doi:10.1111/codi.70137)

Preoperative Anastomotic Evaluation Prior to Ileostomy Closure: A Five-Year UK Survey, Systematic Review, and Meta-Analysis

D. Atraszkiewicz [1], T Shakir [2,3], C. Harrington [3], P. Bassett [4], B. Soile [3], H. Mukhtar [2,3]

Supplement 4 — Meta-Analysis Data

Figure 1: Forest plot showing analysis of the sensitivity and specificity of computed tomography (CT) in identifying anastomotic leaks and abscesses.

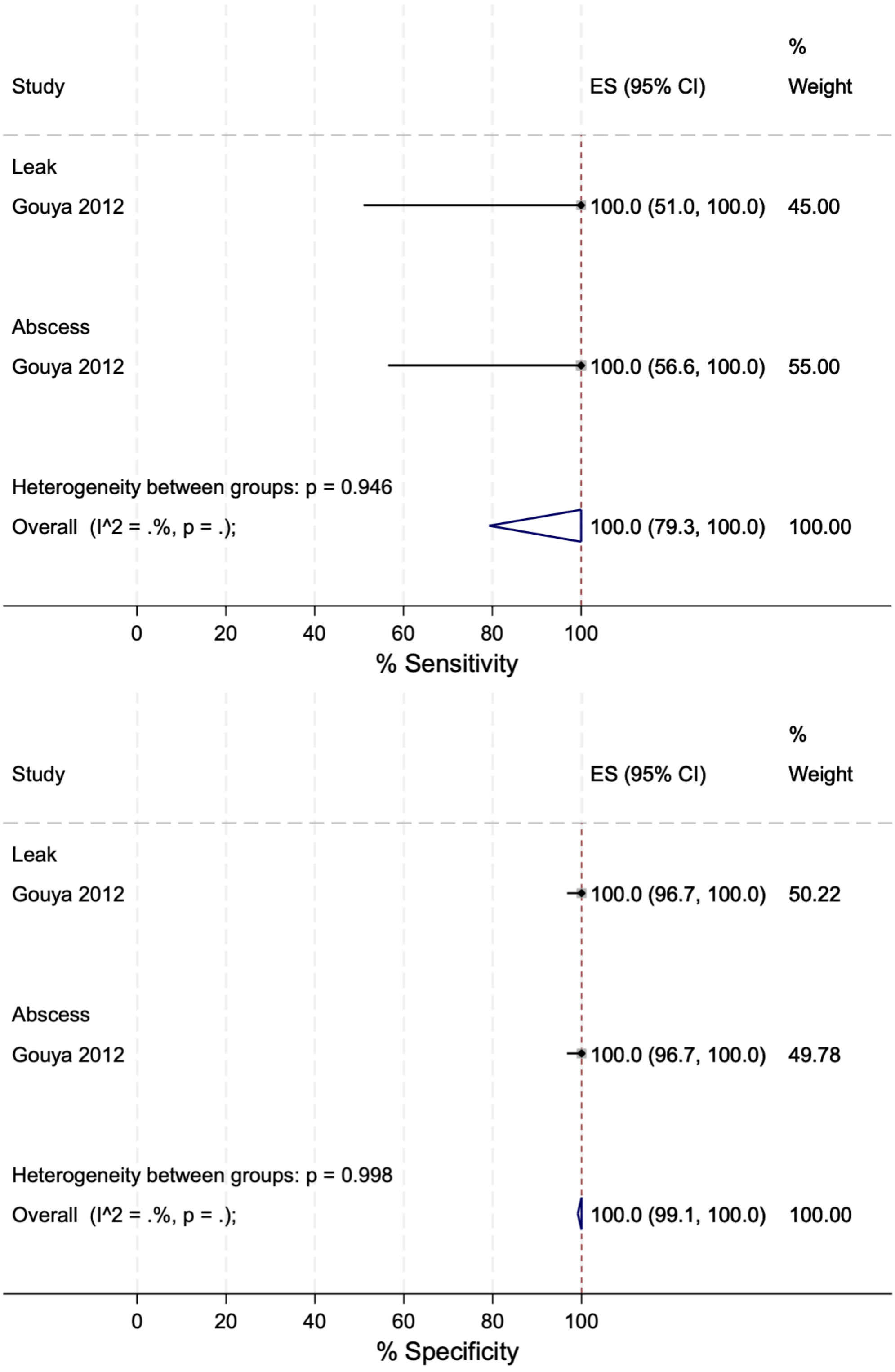

Figure 2: Forest plot showing analysis of the sensitivity and specificity of digital rectal examination (DRE) in identifying anastomotic leaks and strictures.

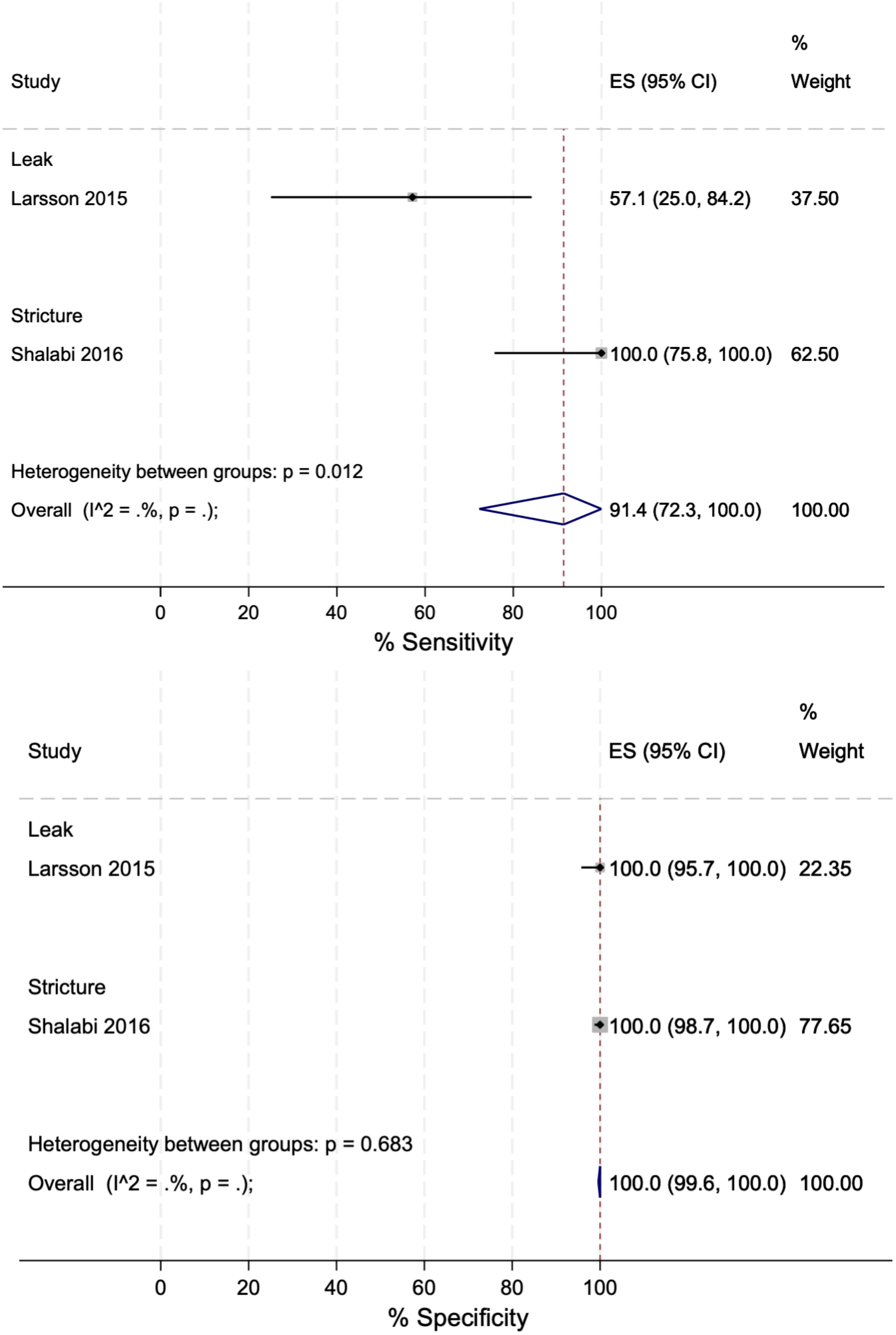

Figure 3: Forest plot showing analysis of the sensitivity and specificity of small bowel follow through (SBFT) in identifying anastomotic leaks and abscesses.

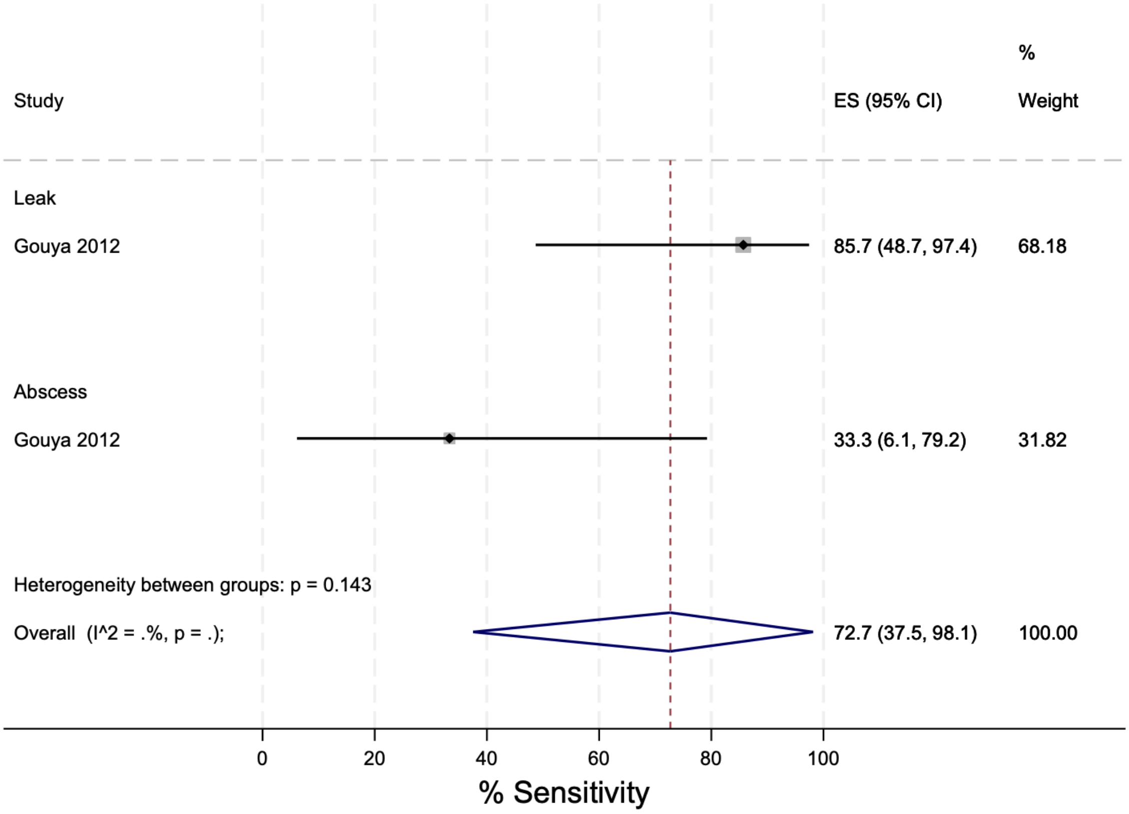

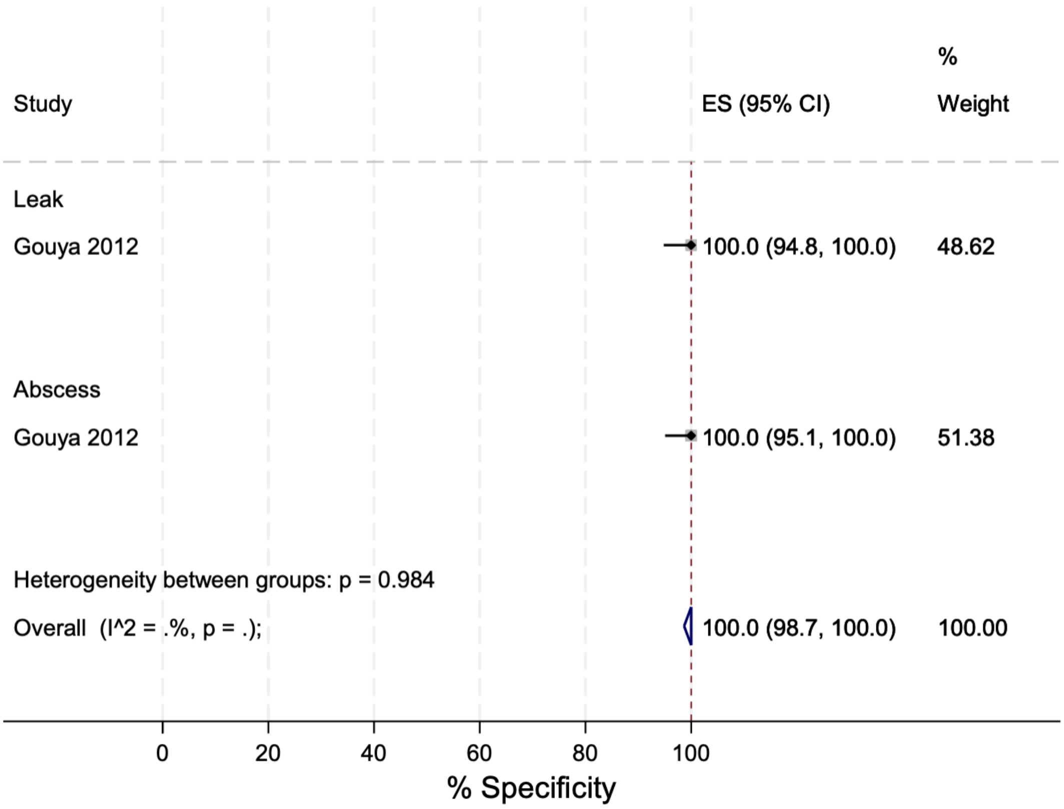

Figure 4: Forest plot showing analysis of the sensitivity and specificity of rigid proctoscopy in identifying strictures.

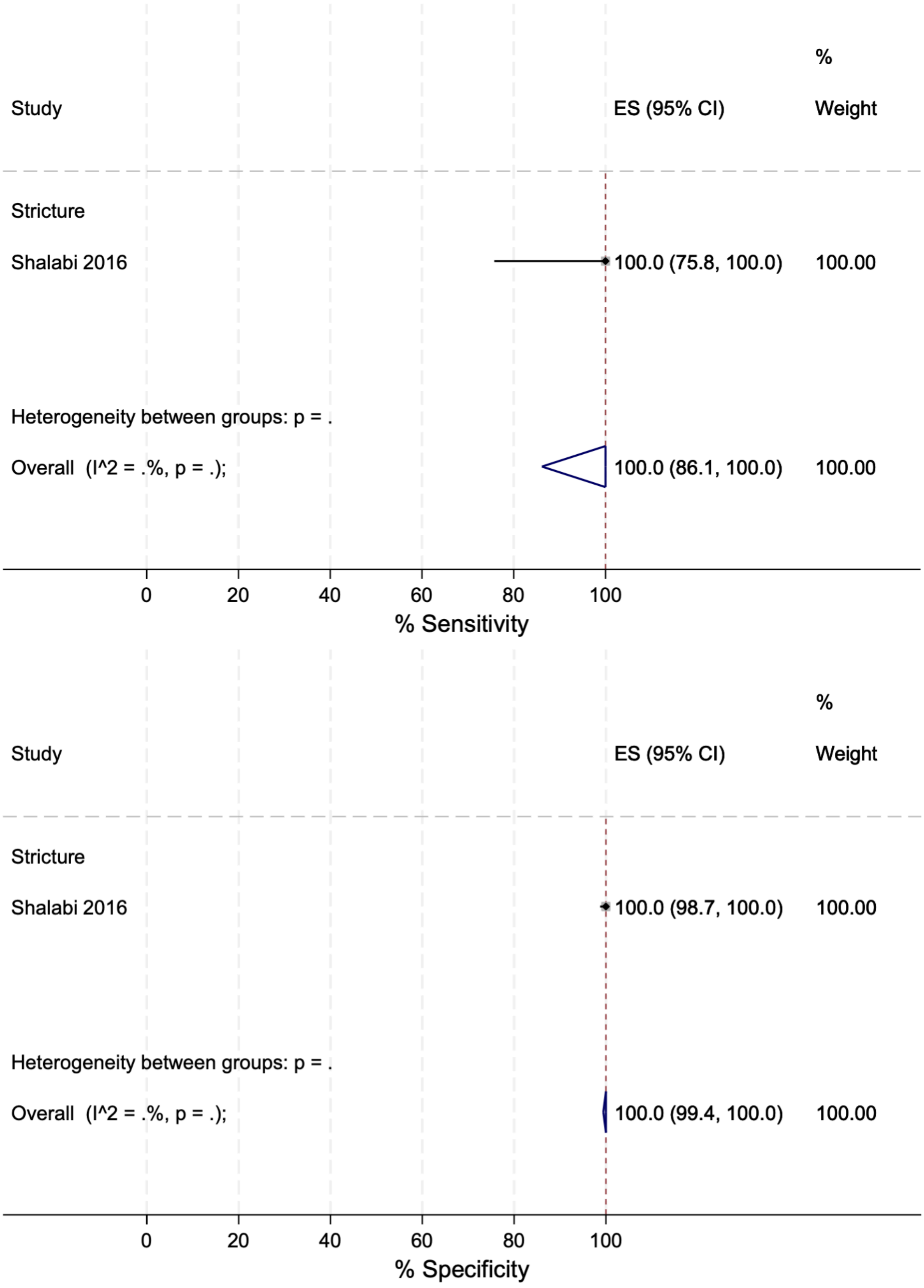

Supplement: Supplementary file 4 — Appendix S4. [file CODI-27-0-s001.pdf]
